# Generation of human induced pluripotent stem cell lines carrying heterozygous PLN mutation from dilated cardiomyopathy patients

**DOI:** 10.1016/j.scr.2022.102855

**Published:** 2022-07-11

**Authors:** Arianne Caudal, Gema Mondejar-Parreño, Carlos D. Vera, Damon R. Williams, Sushma P. Shenoy, David Liang, Joseph C. Wu

**Affiliations:** a Stanford Cardiovascular Institute, Stanford University School of Medicine, Stanford, CA 94305, USA; b Cardiovascular Medicine, Stanford University School of Medicine, Stanford, CA 94305, USA; c Department of Radiology, Stanford University School of Medicine, Stanford, CA 94305, USA

**Keywords:** iPSC, Stem cell, Dilated cardiomyopathy, Phospholamban

## Abstract

Familial dilated cardiomyopathy (DCM) is among the most prevalent forms of inherited heart disease. Here, two human-induced pluripotent stem cell (iPSC) lines were generated from peripheral blood mononuclear cells (PBMCs) from DCM patients carrying different mutations in the phospholamban encoding-gene (PLN). Both iPSC lines exhibited normal morphology, karyotype, pluripotency marker expression, and differentiation into the three germ layers. These patient-specific iPSC lines serve as valuable in vitro models for DCM pathology caused by PLN mutations.

## 1. Resource table

**Table:** 

Unique stem cell lines identifier	1. SCVIi049-A
	2. SCVIi050-A
Alternative name(s) of stem cell lines	1. SCVIi049-A / SCVI104
	2. SCVIi050-A / SCVI2486
Institution	Stanford Cardiovascular Institute, Stanford, CA, US
Contact information of distributor	Joseph C. Wu, joewu@stanford.edu
Type of cell lines	iPSC
Origin	Human
Additional origin info required for human ESC or iPSC	Age: 44 (SCVIi049-A) and 30 (SCVIi050-A)
	Sex: male
	Ethnicity if known: Not Hispanic or Latino
Cell Source	Fibroblast (SCVIi049-A), PBMC (SCVIi050-A)
Clonality	Clonal
Method of reprogramming	Nonintegrating Sendai virus expression of human OCT4, SOX2, KLF4, and c-MYC
Genetic Modification	Yes
Type of Genetic Modification	Spontaneous mutation
Evidence of the reprogramming transgene loss (including genomic copy if applicable)	RT-qPCR
Associated disease	Dilated cardiomyopathy (DCM)
Gene/locus	PLN (6q22.31)
	SCVIi049-A: heterozygous PLN (c.25C > T)
	SCVIi050-A: heterozygous PLN (c.40_42delAGA)
Date archived/stock date	SCVIi049-A: 09/10/2019
	SCVIi050-A: 12/03/2021
Cell line repository/bank	https://hpscreg.eu/cell-line/SCVIi049-A
	https://hpscreg.eu/cell-line/SCVIi050-A
Ethical approval	The generation of the lines was approved by the Administrative Panel of Human Subjects Research (IRB) under IRB #29904 “Derivation of Human Induced Pluripotent Stem Cells”

## 2. Resource utility

Patients carrying pathogenic (c.40_42delAGA) and likely pathogenic (c.25 C>T) mutations in the PLN gene developed dilated cardiomyopathy (DCM). Generation of iPSC lines carrying these mutations provides an unlimited source for differentiation into cardiac cell types (e.g., cardiomyocytes, endothelial cells, fibroblasts), thus providing an excellent tool for in vitro modeling of DCM pathogenesis, testing of candidate therapies, and advancement of personalized medicine (see Table 1).

## 3. Resource details

Dilated cardiomyopathy (DCM), with a prevalence of nearly 1:2,500 people, is the most common cause of heart failure after coronary artery disease and the leading indication for heart transplantation (Maron et al., 2006). Clinical hallmarks of DCM include contractile dysfunction and thinning of the myocardium. Intracellular Ca2+ handling is the central coordinator of cardiac contraction and relaxation. Phospholamban, encoded by the PLN gene, is an abundant, 52 amino acid transmembrane SR phosphoprotein that regulates cardiomyocyte calcium handling as the primary inhibitor of sarco/endoplasmic reticulum Ca2+-ATPase (SERCA) (Schmitt et al., 2003). Several disease variants in the PLN gene have been described in heart failure, but no specific therapies exist beyond standard heart failure treatments or heart transplantation (Eijgenraam et al., 2020). The underlying mechanisms of PLN mutations in DCM remain incompletely understood. Using small animal modeling to study mutation-specific studies is historically a laborious, expensive, time-consuming strategy, taking years before the results of a single treatment may be evaluated (Eijgenraam et al., 2020). The advent of iPSC technology makes in vitro modeling of cardiac diseases possible. Here, cardiovascular cell types derived from patient-specific iPSCs with mutations in PLN present a valuable research opportunity to model DCM disease mechanisms.

**Fig. 1.**
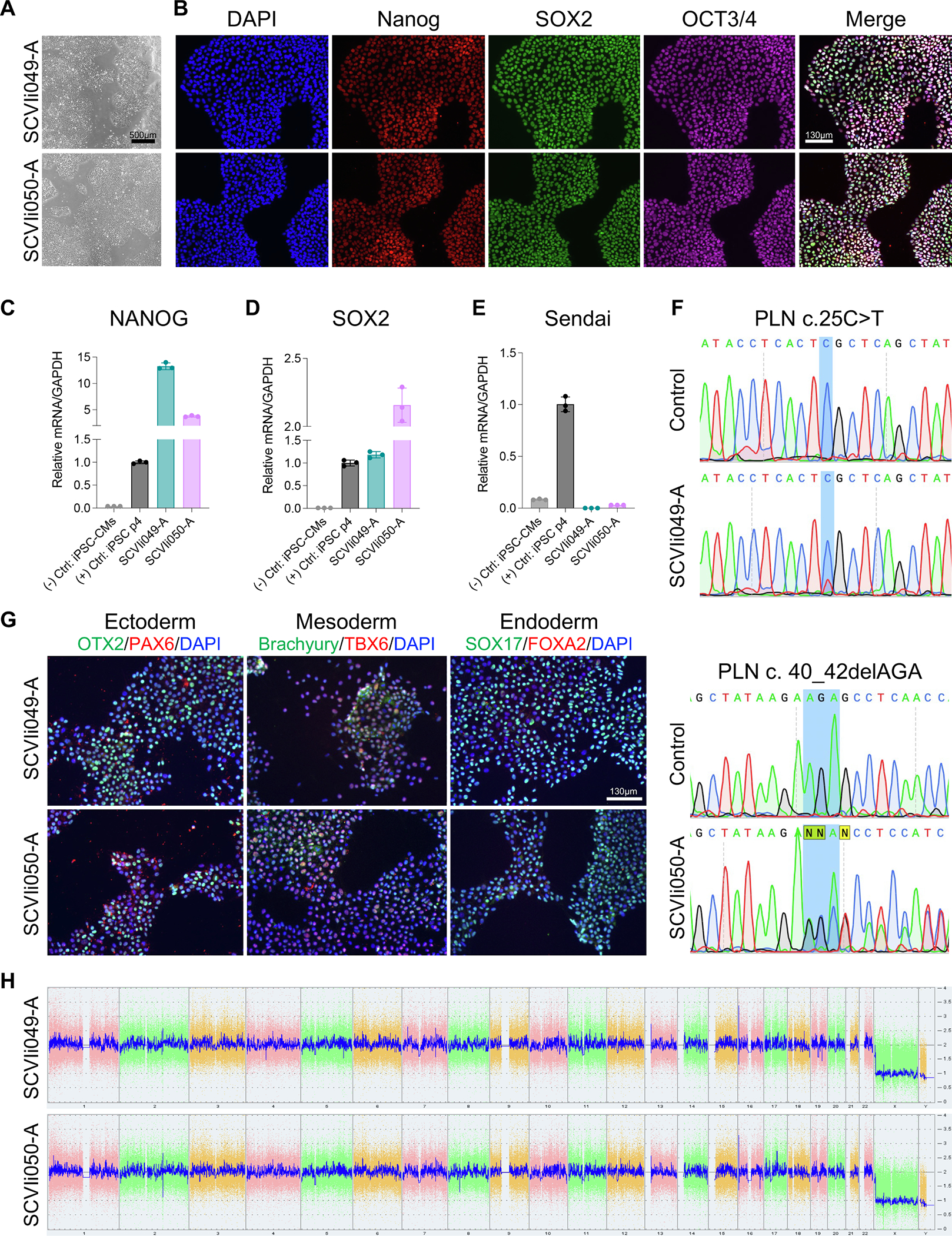
Characterization of patient-derived iPSC lines with c.25C>T and c.40_42delAGA mutations in *PLN*.

We derived two human iPSC lines (SCVIi049-A and SCVIi050-A) from peripheral blood mononuclear cells (PBMCs) and fibroblasts of two patients carrying variants in the PLN gene, including a 44-year-old East Asian male (SCVIi049-A, c.25 C>T encoding p.Arg9Cys, likely pathogenic), and a 30-year-old Caucasian male (SCVIi050-A, c.40_42delAGA encoding p.Arg14del, pathogenic) (Resource Table). Reprogramming of somatic donor cells to iPSCs was conducted using a non-integrating Sendai virus containing the four Yamanaka factors described previously (Liu et al., 2021). Both iPSC lines showed typical morphology (Fig. 1A, Table 1). SCVI049-A and SCVIi050-A demonstrated high expression of pluripotency markers, OCT3/4, NANOG, and SOX2 detected by immunofluorescence (Fig. 1B). The expression of pluripotency markers was confirmed by reverse transcription-quantitative polymerase chain reaction (RT-qPCR). Both SCVIi049-A and SCVIi050-A had comparable SOX2 and NANOG expression levels to the widely used positive control line, SCVI15 (Sun et al., 2012), but expressed much higher than iPSC-derived cardiomyocytes (iPSC-CMs) derived from SCVI15 (Fig. 1C–D). Furthermore, expression of the non-integrating Sendai virus, present at low passage numbers (SCVI15, p4), was absent in SCVIi049-A (p17) and SCVIi050-A (p20) measured by RT-qPCR (Fig. 1E).

The heterozygous mutations of both iPSC lines were confirmed by Sanger sequencing (Fig. 1F). Short tandem repeat (STR) analysis confirmed that both SCVIi049-A and SCVIi050-A demonstrated overlapping profiles with their respective donor somatic cells (Submitted in archive with journal). Additionally, both iPSC lines could differentiate into all three – ectoderm, mesoderm, and ectoderm – germ layers visualized by immunocytochemistry (Fig. 1G). SCVIi049-A and SCVI050-A had normal karyotype results assessed by the KaryoStat™ assay (Fig. 1H). Both iPSC lines were mycoplasma-negative (Supplemental Fig. 1A).

## 4. Materials and methods

### 4.1. Reprogramming

Peripheral blood mononuclear cells (PBMCs) were isolated from patients’ blood by Percoll^R^ gradient separation. PBMCs were purified and replated as previously described (Liu et al., 2021). Briefly, PBMCs were cultured in 1 ml of Stem-Pro™-34 medium (100 ng/ml FLT3, 20 ng/ml IL-6, 20 ng/ml EPO, 20 ng/ml IL-3, and 100 ng/ml SCF). PBMCs were resuspended in 300ul of Stem-Pro™-34 medium and transduced with Sendai virus reprogramming cocktail (CytoTune®-iPSC Sendai Reprogramming Kit). After 24 h, cells were replated, and the medium was replaced every two days. On Day 7, 1 ml of supplemented StemMACS™ iPSC-Brew XF medium (Miltenyi Biotec) was added on top of Stem-Pro™-34 medium. On Day 8, the medium was replaced completely with StemMACS™ iPSC-Brew XF medium. Fresh StemMACS™ iPSC-Brew XF medium was replaced on Days 10–15 when colonies appeared.

### 4.2. Cell culture

Patient-derived iPSCs were cultured in StemMACS iPS-Brew XF medium. Rock inhibitor (10uM, Y27632 Selleck Chemicals) was added up to 24 h after passage. Medium was replaced every two days until confluency. Cells were maintained in a 37 °C incubator with 5% CO_2_ and 20% O_2_.

### 4.3. Karyotyping

Patient-derived iPSCs were analyzed using the KaryoStat™ assay (ThermoFisher Scientific) at p10 (SCVIi049-A) and p8 (SCVIi050-A).

### 4.4. Trilineage differentiation

STEMdiff™ Trilineage Differentiation Kit (STEMCELL Technologies #05230) was used to induce differentiation into endoderm and ectoderm. Mesoderm differentiation was induced with RPMI + glucose medium with B27 minus insulin. Differentiation was performed at p10 (SCVIi049-A) and p8 (SCVIi050-A).

### 4.5. Immunofluorescence staining

At room temperature, cells were fixed in 4% paraformaldehyde, then permeabilized with 50 ug/ml digitonin (Sigma Aldrich #D141) for 10 min each. Cells were incubated with a blocking solution (1% BSA) for 30 min. Cells were incubated with primary antibodies (Table 2) overnight at 4 °C. The following day, cells were washed 3 times. Cells were incubated in secondary antibodies (Table 2) for 30 min at room temperature, then washed 3 times. Nuclei were stained with Molecular Probes NucBlue (ThermoFisher Scientific #R37606) for 10 min at room temperature. Cells were washed 3 times, then imaged using a confocal light microscope. Immunostaining was carried out at p16 (SCVIi049-A) and p19 (SCVIi050-A).

### 4.6. RT-qPCR

RNA was extracted using the Direct-zol™ RNA Miniprep Kit (ZYMO Research #3R2061). To generate cDNA, iScript™ cDNA Synthesis Kit (BioRad #1708891) was used as follows: 5 min at 25 °C, 20 min at 46 °C, and 1 min at 95 °C. Expression of SOX2, NANOG, and SEV was amplified using commercial primers (Table 2) and TaqMan™ Gene expression Assay (Applied Biosystems™ #4444556).

### 4.7. Short tandem repeat analysis

Genomic DNA (gDNA) from fibroblasts (SCVI049-A), PBMCs (SCVIi050-A), and iPSCs were purified using DNeasy Blood & Tissue Kit (Qiagen). STR analysis was performed using CLA Identifier™ Plus and Identifier™ Direct PCR Amplification Kits (Thermo Fisher) by the Stanford PAN Facility.

### 4.8. Sanger sequencing

PCR primers were designed to flank PLN mutations (Table 2) and used to amplify the genomic region using Q5® Hot Start High-Fidelity DNA Polymerase (New England BioLabs). The PCR reaction was performed as follows: 98 °C for 5 sec, 62 °C for 10 sec, 72 °C for 20 sec for 35 cycles. PCR products were purified using QIAquick Purification Kit (Qiagen) and sent to the Stanford PAN facility.

### 4.9. Mycoplasma detection

Mycoplasma contamination was evaluated using a MycoAlert Detection Kit (Lonza #LT07–318) at p17 (SCVIi049-A) and p20 (SCVIi050-A).

## Supplementary Material

1

## Figures and Tables

**Table 1:** Characterization and validation.

Classification	Test	Result	Data

Morphology	Photography bright field	Normal	Fig. 1A
Phenotype	Qualitative analysis	Positive expression of pluripotency markers by immunocytochemistry: NANOG, SOX2, and OCT3/4	Fig. 1B
Genotype	Karyotype (G-banding) and resolution	Karyostat™ Assay, resolution 1–2 Mb: Normal karyotype 46, XY for both lines.	Fig. 1H
Identity	Microsatellite PCR (mPCR) or STR analysis	Not performed	N/A
		22 loci tested, 100% identical	Submitted in archive with journal
Mutation analysis (IF APPLICABLE)	Sequencing	SCVIi049-A: heterozygous c.25C > T	Fig. 1F
		SCVIi050-A: heterozygous c.40_42delAGA	
	Southern blot OR WGS	Not performed	Not performed
Microbiology and virology	Mycoplasma	Mycoplasma testing by luminescence: Negative (p10 and above)	Supplemental Fig. 1A
Differentiation potential	Embryoid body formation or Teratoma formation or Scorecard or directed differentiation	Directed differentiation, positive expression of germ layer markers	Fig. 1G
List of recommended germ layer markers	Expression of these markers has to be demonstrated at mRNA (RT PCR) or protein (IF) levels, at least 2 markers need to be shown per germ layer	Positive expression of germ layer markers:	Fig. 1G
		Ectoderm: Pax6, Otx2	
		Mesoderm: Brachyury, Tbx6	
		Endoderm: Sox17, Foxa2	
Donor screening (OPTIONAL)	HIV 1 + 2 Hepatitis B, Hepatitis C	Not performed	Not performed
Genotype additional info (OPTIONAL)	Blood group genotyping	Not performed	Not performed
	HLA tissue typing	Not performed	Not performed

**Table 2:** Reagents details.

	Antibodies used for immunocytochemistry/flow-cytometry			
	Antibody	Dilution	Company Cat #	RRID

Pluripotency Marker	Mouse IgG2b kAnti-OCT-3/4	1:100	Santa Cruz Biotechnology Cat# sc-5279	RRID: AB_628051
Pluripotency Marker	Rabbit Anti-NANOG	1:100	Protein Tech Cat# 142951-1-AP	RRID: AB_1607719
Pluripotency Marker	Mouse IgG1 kAnti-SOX2	1:100		RRID: AB_10842165
			Santa Cruz Biotechnology Cat# sc-365823	
Differentiation Marker (Ectoderm)	Goat Anti-OTX2	1:200	R&D Systems Cat#963273	RRID: AB_2157172
Differentiation Marker (Ectoderm)	Rabbit Anti-PAX6	1:200	Thermo Fisher Scientific Cat#42-6600	RRID: AB_2533534
Differentiation Marker (Endoderm)	Goat Anti-SOX17	1:200	R&D Systems Cat#963121	RRID: AB_355060
Differentiation Marker (Endoderm)	Rabbit Anti-FOXA2	1:250	Thermo Fisher Scientific Cat#701693	RRID: AB_2576439
Differentiation Marker (Mesoderm)	Goat Anti-Brachyury	1:200	R&D Systems Cat#963427	RRID: AB_2200235
Differentiation Marker (Mesoderm)	Rabbit Anti-TBX6	1:200	Thermo Scientific Cat#PA5-35102	RRID: AB2552412
Secondary Antibody	Alexa Fluor 488 Goat Anti-Mouse IgG1	1:1000	Thermo Fisher Scientific #A-21121	RRID: AB_2535764
Secondary Antibody	Alexa Fluor 647 Goat Anti-Mouse IgG2b	1:250	Thermo Fisher Scientific #A21242	RRID: AB_2535811
Secondary Antibody	Alexa Fluor 555 Goat Anti-Rabbit IgG (H+L)	1:500	Thermo Fisher Scientific #A-21428	RRID: AB_141784
Secondary Antibody	Alexa Fluor 488 Donkey Anti-Goat IgG (H+L)	1:1000	Thermo Fisher Scientific #A-11055	RRID: AB_2534102
	Primers Target	Size of band	Forward/Reverse primer (5′-3′)	

Genotyping	SCVIi049-A: c.25 C>T	376 bp	F: TTTTACATTCCAGGCTACCTAAAAG	
			R: TCTACTCAGGAAGTGGTCTGT	
Genotyping	SCVIi050-A: c.40_42delAGA	376 bp	F: TTTTACATTCCAGGCTACCTAAAAG	
			R: TCTACTCAGGAAGTGGTCTGT	
Sendai virus plasmid (RT-qPCR)	Sendai virus genome	181 bp	Mr042698800_mr	
Pluripotency markers (RT-qPCR)	SOX2	258 bp	Hs04234836_s1	
Pluripotency markers (RT-qPCR)	NANOG	327 bp	Hs02387400_g1	
Housekeeping genes (RT-qPCR)	GAPDH	471 bp	Hs02786624_g1	
