# Supplementary figures and images for: Generation of human induced pluripotent stem cell lines carrying heterozygous PLN mutation from dilated cardiomyopathy patients
Source: Stem Cell Res. Author manuscript; Available in PMC 2022 Nov 23. (PMC9681708; doi:10.1016/j.scr.2022.102855)

**A**      Mycoplasma

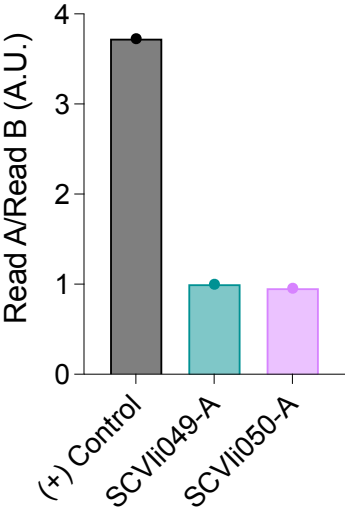

Supplement: 1 [file NIHMS1827839-supplement-1.pdf]
